# Electromagnetic validation of a multichannel coplanar four-electrode bioimpedance sensing platform for perfused 3D tumor-on-chip systems

**DOI:** 10.2478/joeb-2026-0015

**Published:** 2026-10-12

**Authors:** Félix Royer, Thomas Meynard, Robin Houssier, Jérôme Vicogne, Ørjan G. Martinsen, Isabelle Van Seuningen, Vincent Senez

**Affiliations:** Univ. Lille, Inserm, CHU Lille, CNRS, U1366-UMR9020 – CRCLille, F-59000 Lille, France; Univ. Lille, CNRS, Inserm, CHU Lille, IPL, U1019-UMR9017– CIIL, F-59000 Lille, France; Department of Physics, University of Oslo, Oslo, Norway; Department of Clinical and Biomedical Engineering, Oslo University Hospital,Norway

**Keywords:** Electrical impedance spectroscopy, tumor-on-chip, sensitivity field / lead-field analysis, 3D hydrogel culture, coplanar four-electrode

## Abstract

Three-dimensional tumor-on-chip systems better reproduce the spatial organization of tumor cells within extracellular-matrix-based microenvironments than conventional two-dimensional cultures, yet their integration with quantitative, non-destructive monitoring tools remains limited. Electrical bioimpedance provides a label-free approach for real-time assessment of living 3D tissues, but its implementation in perfused millimeter-scale microfluidic environments requires rigorous electrical and geometrical validation.

In this study, we designed and electromagnetically validated an integrated coplanar four-electrode bioimpedance sensor embedded within a perfused 3D tumor-on-chip platform compatible with collagen-based hydrogels. Three-dimensional finite-element simulations were used to compute sensitivity fields and quantify the effective measurement volume. Calibration with conductivity standards showed quantitative agreement between experimental measurements and numerical predictions, with relative deviations below 10%, enabling reliable extraction of the cell constant. Chamber height was identified as the dominant geometrical parameter affecting impedance magnitude, whereas lateral misalignment had limited impact. Temperature-dependent conductivity changes induced impedance drifts of up to 25% during thermal equilibration, highlighting the need for strict temperature control.

Acellular collagen I hydrogels exhibited concentration-dependent impedance variations, while increased gel loading before chamber closure modified impedance, likely through matrix compression and reorganization. These results demonstrate that the extracellular matrix significantly contributes to the measured electrical response. In static 3D cultures, embedding pancreatic cancer cells within collagen I produced a reproducible decrease in impedance magnitude at 10 kHz compared with acellular controls, confirming a measurable cell-dependent baseline shift. Under continuous perfusion, gradual cellular evolution did not generate a reproducible impedance trajectory, whereas Triton X-100-induced cell disruption increased impedance toward the acellular collagen I baseline.

Overall, this study demonstrates that coplanar impedance sensing can detect cell-dependent baseline differences and strong endpoint perturbations in millimeter-scale 3D tumor models. However, dynamic impedance variations under perfusion remain influenced by matrix, thermal, geometrical, and conductive-pathway contributions, emphasizing the need for careful calibration before biological interpretation.

## Introduction

Solid tumors are complex and heterogeneous tissues composed of cancer cells, non-malignant stromal cells, immune cells, and vascular components embedded within an extracellular matrix (ECM) and permeated by interstitial fluids. Beyond genetic and biochemical alterations, tumor progression and therapeutic response are strongly influenced by biophysical factors such as ECM composition, solid stress, interstitial fluid pressure and interstitial flow [1,2,3]. Accurately reproducing these features *in vitro* remains a major challenge and is critical for improving the predictive value of preclinical cancer models.

Three-dimensional (3D*) in vitro* tumor models, including multicellular spheroids and hydrogel-based cultures, have emerged as more structurally and microenvironmentally relevant alternatives to conventional two-dimensional monolayers. By enabling cell-cell and cell-ECM interactions as well as spatial organization and gradients, 3D models better recapitulate tumor architecture and phenotype [4,5,6]. However, most 3D culture systems remain static and therefore fail to reproduce perfusion-driven mass transport, including nutrient delivery, waste removal, and drug penetration, which are key features of solid tumor microenvironments.

Microfluidic tumor-on-chip (ToC) platforms have been developed to overcome these limitations by enabling dynamic perfusion under well-controlled conditions. Operating in the laminar flow regime, such systems allow precise control over mass transport, mechanical cues and microenvironmental parameters, making them particularly suitable for modelling solid tumors under structurally and transport-relevant dynamic conditions [7,8,9]. Recent advances in hybrid microfabrication strategies combining soft lithography and high-resolution 3D printing have further expanded the architectural complexity and accessibility of ToC platforms for dynamic 2D and 3D tissue culture [10–11].

Despite these advances, monitoring cell viability and proliferation in dynamic 3D ToC models remains a significant methodological challenge. Conventional viability assays, including metabolic activity measurements, membrane integrity assays and fluorescent live/dead staining, are typically destructive, endpoint-based and incompatible with continuous monitoring [12,13,14]. In addition, optical readouts suffer from intrinsic limitations in thick 3D tissues due to light scattering, limited penetration depth and heterogeneous signal distribution. Although advanced imaging strategies can partially mitigate these issues, they often require dedicated live-imaging equipment to be immobilized for extended periods, making them costly, experimentally restrictive, and difficult to scale for long-term monitoring of multiple dynamic culture conditions [15,16].

Electrical bioimpedance has long been recognized as a powerful tool for monitoring cellular behavior, including adhesion, proliferation and morphological changes, through non-invasive and label-free measurements, i.e., without requiring exogenous fluorescent or molecular labels [17]. Bioimpedance measurements probe the opposition of biological materials to an applied alternating current and are sensitive to the electrical properties of cells, extracellular fluids and ECM. Within frequency ranges where electrode-polarization effects are limited and cellular membrane contributions remain measurable, impedance is strongly influenced by cell density, membrane integrity and tissue organization, making it particularly relevant for monitoring viability and proliferation in living tissues [18,19,20].

More recently, impedance spectroscopy has been extended to three-dimensional epithelial tissue models, where it provides access to collective electrical properties emerging from cell-cell and cell-matrix interactions [21]. Impedance-based approaches have also been applied to drug testing in 3D *in vitro* tissues using microfabricated platforms [22], as well as to advanced electrode geometries enabling electrical interrogation of three-dimensional cell cultures for anticancer drug assessment [23,24]. However, most of these studies rely on static conditions or simplified architectures and do not fully address the challenges associated with dynamic perfusion and volumetric tumor-on-chip systems. In particular, the electromagnetic validity and volumetric sensitivity of coplanar impedance configurations in perfused millimeter-scale 3D systems require rigorous definition and experimental characterization.

In this work, we combine a perfused 3D ToC platform with an integrated coplanar four-electrode bioimpedance sensor to evaluate the feasibility, quantitative validity, and physical constraints of label-free impedance measurements in collagen-based 3D cancer cultures. The four-electrode architecture was implemented to reduce electrode polarization effects, while numerical sensitivity-field simulations were used to characterize the effective measurement volume within millimeter-scale hydrogel constructs. Rather than assuming impedance to be a direct proxy for cell number or viability, we systematically examined the respective contributions of electrode configuration, chamber geometry, temperature, extra-cellular matrix properties, and embedded pancreatic cancer cells. This framework was used to assess both the ability of coplanar impedance sensing to detect cell-dependent baseline differences and the limitations encountered during time-resolved monitoring under continuous perfusion.

This paper is organized as follows. The Materials and Methods section describes the microfluidic platform, electrode design and fabrication, impedance measurement protocol, numerical modelling, and biological models. The Results section first reports the electrical, geometrical, thermal, and matrix-related validation of the system, before evaluating the detection of fluorescent pancreatic cancer cells embedded in collagen I hydrogels under static and perfused conditions. The Discussion then examines how sensitivity-field distribution, conductive volume, matrix properties, and perfusion-related effects constrain the interpretation of dynamic impedance measurements in 3D ToC systems.

## Materials and methods

### Manufacturing and integration of the tumor-on-chip system

#### Tumor-on-chip device architecture

The ToC device consists of a microfluidic assembly comprising (i) a glass substrate bearing the microelectrode array, (ii) a polydimethylsiloxane (PDMS) bottom part defining the culture chambers and microfluidic channels, (iii) a rigid cap setting the culture chamber height, and (iv) a custom mechanical closing system composed of a reinforced 3D-printed top plate, an aluminum wedge, and an aluminum bottom closing plate, designed to accommodate the protruding PCB while ensuring reproducible sealing and mechanical stability during perfusion (Supplementary Figure S1).

The device comprises eight parallel culture chambers, each interfaced with eight electrodes, resulting in a total of 64 individually addressable electrodes. This architecture enables multiplexed impedance measurements across the eight culture chambers using the 64-channel impedance measurement system.

All components were designed using Autodesk Fusion 360, version 2.0.20256 x86_64 (Autodesk, San Francisco, CA, USA) computer-aided design (CAD) software and exported in stereolithography (STL) format for manufacturing. STL files intended for resin printing were prepared using CHITUBOX Basic, version 2.1. The aluminum lower closing plate and wedge were fabricated by computer numerical control (CNC) milling using a Datron Neo milling machine (Datron Neo, Datron, Annecy, France) from an aluminum plate (RS Components Ltd., Corby, UK, Metal Sheet reference 188-321) using micro-end mills (Datron, Annecy, France, Single Flute reference 0068020E). The rigid upper cap was fabricated by stereolithography using a biocompatible resin (ATOME3D, Lespinasse, France, Bio-Med Clear LIQCREATE) using an LCD 3D printer (Phrozen, Hsinchu, Taiwan, Sonic Mini 8K).

Rigid caps incorporating multiple predefined heights were used to generate distinct culture chamber heights on a single device for geometrical sensitivity studies. The design and fabrication of the 3D-printed rigid caps are described in detail elsewhere [10].

#### PDMS microfluidic bottom fabrication and bonding

The PDMS microfluidic bottom was fabricated by replica molding using Sylgard 184 Silicone Elastomer (Darwin Microfluidics, Paris, France) mixed with its curing agent at a 10:1 (w/w) ratio. The mixture was degassed for 30 min, poured into master molds closed using a set of 3D-printed rings holding a 200 μm thick polyvinyl chloride (PVC) sheet (Fellowes Brands, Itasca, IL, USA; reference CRC53761) to ensure a smooth surface once demolded, degassed for an additional 20 min, and cured in an oven (Virtus GmbH, Hamm, Germany; model 5695TD) at 70 °C for 3 h.

After curing, PDMS parts were demolded using a scalpel and tweezers and washed for 3 min in isopropyl alcohol (IPA) using an ultrasonic cleaner. Bonding to the glass substrate, 76 mm × 26 mm (Trajan Scientific and Medical, Milton Keynes, UK, reference 471242000) was performed after oxygen plasma activation (Harrick Plasma, Ithaca, USA; PDC-002-CE) for 60 s, followed by a hot-plate step at 120 °C for 2 min.

#### Microelectrode array design

Each culture chamber was associated with eight coplanar electrodes, allowing flexible selection of current-carrying and potential-sensing electrode pairs for four-electrode transfer-impedance measurements. Alignment ruler patterns consisting of 10 μm wide digits with a 100 μm pitch were integrated on the electrode substrate to quantify alignment accuracy between PDMS and electrode layers during assembly.

These ruler patterns were used to quantify lateral alignment errors between the PDMS microfluidic layer and the electrode-bearing glass substrate in both x- and y-directions after bonding. The theoretical position of the ruler relative to the culture chamber walls was predefined during electrode design, enabling post-bonding measurement of alignment offsets. X-direction misalignment was extracted directly from ruler digits, while y-direction misalignment was quantified using image-based proportional scaling from optical micrographs.

#### Electrode fabrication on glass substrate

Microelectrodes were fabricated on glass substrates using a bilayer lift-off photolithography process. Glass substrates were cleaned in piranha solution (3:1 (v/v) sulfuric acid to hydrogen peroxide) for 10 min, rinsed thoroughly in deionized water, and dehydrated at 240 °C for 10 min. Substrates were then placed in acetone in an ultrasonic cleaner for 10 min, rinsed, and dehydrated again.

A 220 nm LOR-3A layer (Micro resist technology GmbH, Berlin, Germany) was spin-coated using a Laurell spin coater (Laurell Technologies Corporation, Lansdale, USA; model EDC-650-8B) (10 s at 800 rpm, acceleration 1000, followed by 60 s at 3000 rpm) and soft-baked for 5 min at 190 °C. A second 1.5 μm S1813 photoresist layer (Micro resist technology GmbH, Berlin, Germany) was deposited using the same spin sequence and soft-baked for 1 min 20 s at 110 °C. UV exposure (390 nm) was performed using a μPG 501 maskless lithography system (Heidelberg Instruments Mikrotechnik GmbH, Heidelberg-Wieblingen, Germany) for 80 ms, followed by development for 45 s in MEGAPOSIT MF-21A developer (MicroChemicals GmbH, Ulm, Germany).

A Ti/Pt bilayer (20/100 nm) was deposited by electron-beam physical vapor deposition using a Tornado 406E system (Polyteknik AS, Ostervra, Denmark). Lift-off was achieved by alternating three cycles of acetone and developer baths (2 min each), followed by rinsing and dehydration at 200 °C for 10 min.

#### Electrical interfacing and reflow soldering

Electrical interfacing between the microelectrode substrate and the impedance measurement system was achieved using a custom-designed printed circuit board (PCB) (Sciospec Scientific Instruments GmbH, Bennewitz, Germany). The PCB dimensions were 101.61 × 52 mm, with a 65 × 43.2 mm notch allowing the microfluidic device to protrude through the board. The PCB thickness was 1.61 mm and comprised six copper layers alternating with five dielectric cores.

Conductive soldering paste (Chipquik^®^, Niagara Falls, USA; reference SMD291SNL500T4) was applied to the PCB pads through a stencil using a mechanical stretching frame (LPKF Laser & Electronics SE, Garbsen, Germany, model ZelFlex QR). The PCB was aligned with the electrode pads and reflow soldering was performed in a vapor-phase reflow oven (Asscon Systemtechnik-Electronik GmbH, Königsbrunn, Germany, model Asscon Quicky 450 VP) using the following temperature profile: ramp to 150 °C within 90 s, hold at 175 °C for 90 s, ramp to 249 °C within 60 s, followed by cooling to solidify the solder paste. Following assembly, the electrical functionality of the electrode connections was verified during preliminary impedance measurements and calibration using conductivity reference solutions. Channels exhibiting abnormal or unstable electrical responses were excluded from subsequent analyses. No separate quantitative measurement of the individual solder-joint contact impedance was performed.

#### Microfluidic circuit and perfusion hardware

Microfluidic circuits were prepared, washed, and sterilized prior to each experiment. Each circuit comprised a custom-designed 16-in-1 connector interfacing the eight inlet/outlet pairs of the device, a custom-made glass medium reservoir (MOD’VERRE, Grasse, France), silicone tubing or capillaries (1.6 mm inner diameter; Ibidi^®^, Gräfelfing, Germany; reference 10842), 0.5 – 1.6 mm adaptors (Ibidi^®^; reference 10829), and a peristaltic pump (Ismatec™, Glattbrugg, Switzerland; ISM930A). The tubing/capillaries, connectors, and adaptors were coated with a 2 μm-thick Parylene C film using an SCS Labcoter 2 Parylene C deposition system (Specialty Coating Systems, Indianapolis, USA, model PDS 2010). The glass medium reservoirs were not coated. The device caps were also coated with Parylene C after Sudan Black B staining (Sigma-Aldrich, Darmstadt, Germany, reference 380B-1KT). Circuits were primed without the device by connecting them to a running peristaltic pump (Ismatec, Glattbrugg, Switzerland, model IPC-N8 ISM936) to fill the tubing with culture medium prior to device connection. For flow actuation, 0.5 mm tubing (Ibidi, Gräfelfing, Germany, reference 10840) was used in the pump head.

### Experimental settings and impedance measurements

#### Flow rate verification assay

Flow rates were verified using two complementary approaches. For peristaltic pump calibration, flow meters (Fluigent, Le Kremlin-Bicêtre, France, model Flow Sensor M) were placed downstream of the chip. The circuit was filled with 3 mL of water and flow was imposed using multichannel Ismatec peristaltic pumps at nominal flow rates of 3.74, 10, 25, and 50 μL·min^−1^. Actual flow rates were recorded using the flow meters.

For fluorescein bolus assays, an in-line injection port (Ibidi, Gräfelfing, Germany) was fitted upstream of the device and the circuit was filled with 3 mL of deionized water. Fluorescein (Sigma-Aldrich, Darmstadt, Germany) was prepared at a concentration of 0.47 mol·m^−3^. A 100 μL bolus was injected using a 1 mL syringe through a silicone septum with a needle (Terumo, Shibuya, Japan, model Terumo Agani). Flow was imposed using a high-precision multichannel pump (Ismatec, Glattbrugg, Switzerland, model IPC-N8 ISM936), and imaging was performed on a DMI8 microscope (Leica Microsystems, Wetzlar, Germany). Fluorescence intensity time traces were extracted from two regions of interest along the channel to estimate mean flow velocity.

#### Impedance instrumentation and acquisition parameters

Impedance measurements were performed using a 64-channel impedance measurement system (Sciospec Scientific Instruments GmbH, Bennewitz, Germany, model CSX-64). Data acquisition was controlled using custom Python scripts executed in PyCharm Community Edition 2023.1.1 (JetBrains), within a dedicated Python virtual environment to ensure reproducibility.

In the four-electrode configuration, current was injected through the current-carrying (CC) electrode pair, while the voltage difference was measured between a spatially distinct pair of potential-sensing (PU) electrodes. The measured quantity therefore corresponds to a transfer impedance, defined as *Z_t_* = Δ*V*_PU_/*I*_CC_.

The excitation signal was set to 25 mV peak-to-peak to maintain small-signal measurement conditions and minimize electrochemical perturbation at the electrode-electrolyte interface while preserving sufficient signal amplitude. Impedance spectra of liquids and hydrogels were acquired from 500 Hz to 60 kHz. A complete impedance-spectrum acquisition cycle covering all eight culture chambers required approximately 2 min 30 s. This range was selected to include the transition from the low-frequency region affected by electrode polarization to the quasi-resistive response of the conductive medium, while limiting higher-frequency parasitic contributions from the measurement setup. For time-resolved experiments, impedance magnitude was monitored at 10 kHz with a measurement interval of 3 min. The total acquisition duration depended on the experiment and is specified in the corresponding experimental subsections. This frequency was selected within the stable four-electrode impedance plateau observed experimentally above approximately 2 kHz, providing reduced sensitivity to electrode polarization while retaining sensitivity to changes within the culture volume.

#### Electrical calibration using conductivity reference solutions

Electrical calibration of the impedance measurement system was performed using three commercial electrical conductivity standard solutions (20, 30, and 50 mS·cm^−1^; Sigma-Aldrich, Darmstadt, Germany references 51715, 91916, 56255) and culture medium (1X DMEM). The electrical conductivity of each solution was independently measured using a SevenDirect SD23 conductivity meter (Mettler Toledo, Columbus, USA). All measurements were conducted at room temperature (22.3 °C).

Culture chambers were filled with 60 μL of each solution and impedance spectra were acquired over the 500 Hz – 60 kHz frequency range using a four-electrode configuration. The outermost potential-sensing (PU) electrode pair was used for analysis, assuming homogeneous filling of the culture chamber and purely conductive behavior of the reference solutions within the investigated frequency range.

For each conductivity standard, the impedance magnitude was averaged over the 500 Hz – 60 kHz frequency interval. This frequency-averaged value was used because the four-electrode impedance spectra of the reference solutions were quasi-frequency-independent over the investigated range (Fig. 3a), thereby allowing pointwise measurement variability to be reduced. The experimental cell constant was calculated from the measured impedance and the independently measured electrical conductivity of each standard solution according to *k* = *σ* |*Z_mean_*|, where *k* is the cell constant (m^−1^), *σ* is the measured electrical conductivity (S·m^−1^), and ∣ *Z_mean_* | is the frequency-averaged impedance magnitude. Numerical impedance values for the conductivity standards were computed using the three-dimensional COMSOL Multiphysics model under identical excitation and electrode configurations.

#### Temperature-induced impedance drift experiments

Temperature-induced impedance drift experiments were performed using liquid-filled culture chambers in the absence of cells. Each culture chamber was filled with 60 μL of complete 1X DMEM culture medium. Devices were initially equilibrated at ambient laboratory temperature, recorded between 20 and 22 °C, prior to measurements.

Time t = 0 was defined as the moment when the fully assembled device was placed inside a water-humidified incubator set to 37 °C and 5% CO_2_. Impedance magnitude was monitored at a fixed frequency of 10 kHz with a measurement interval of 3 min for a total duration of 120 min.

During temperature drift experiments, impedance was recorded simultaneously on all eight culture chambers of a device. The data corresponds to the mean impedance magnitude across chambers. Individual chamber measurements exhibited comparable temporal dynamics.

The temperature dependence of the electrical conductivity of the culture medium was characterized independently. Conductivity measurements were performed at 25, 30, 35 and 40 °C using a calibrated SevenDirect SD23 conductivity meter (Mettler Toledo, Columbus, OH, USA). For each temperature, the 1X DMEM culture medium was equilibrated under temperature-controlled conditions until thermal stabilization before conductivity measurement.

#### Electrode cleaning and reuse protocol

After impedance measurements, culture chambers were rinsed three times with 100 μL of 1X phosphate-buffered saline (1X PBS). Chambers were then filled with 150 μL of 0.25% (v/v) trypsin solution (Gibco, Paisley, Scotland, reference 25200-056) and incubated for 5 min at 37 °C. After 1X PBS rinsing, chambers were filled with 150 μL of 0.01% (v/v) acetic acid buffer adjusted to pH 3.8 with 0.1N NaOH and 0.1N HCl solutions and incubated for 10 min at room temperature. Chambers were finally rinsed with 1X PBS and dried with compressed air prior to subsequent use.

### Numerical simulations

Numerical simulations were performed using COMSOL Multiphysics^®^ version 5.6 (COMSOL AB, Stockholm, Sweden). 3D geometries of the electrode layouts, culture chambers, and microfluidic circuit components were implemented from CAD designs and discretized using tetrahedral meshes with ‘fine’ or ‘extra-fine’ element sizes, depending on the physics module. Meshes comprised typically 26,000 – 30,000 elements for electrical simulations, with average mesh quality values around 0.6 – 0.7.

#### Electrical simulations and sensitivity field analysis

Sensitivity fields were computed using the AC/DC module (Electric Currents interface) following a reciprocity-based lead-field approach. Two Electric Currents interfaces were defined, corresponding to the reciprocal four-electrode configurations. In each interface, one current-carrying electrode was set to ground (V = 0) and the other was driven with a potential of 25 mV. The resulting total current, *I*_CC_, was calculated by integrating the normal current density over the surface of the driven current-carrying electrode. Electrical insulation boundary conditions were applied to the chamber walls, and the initial potential in the culture volume was set to 0 V.

Unless otherwise stated, the culture chamber was assumed to be filled with a homogeneous medium of electrical conductivity σ = 20 mS·cm^−1^ and relative permittivity ε_r_ = 80. Within the 500 Hz – 60 kHz frequency range and for conductive aqueous media, the impedance response was dominated by conductivity. Permittivity mainly influenced the phase response at the upper end of the spectrum [18].

The four-electrode transfer impedance was calculated as *Z_t_* = Δ*V*_PU/_*I*_CC_, where Δ*V*_PU_ is the potential difference between the selected potential-sensing electrodes and *I*_CC_ is the total current obtained from the numerical solution. The influence of chamber height was evaluated by varying the chamber height from 400 to 700 μm in 50-μm increments, while keeping the electrode geometry and electrical properties unchanged. Lateral misalignment was investigated by varying the relative position of the PDMS chamber with respect to the electrode substrate from −1 to +1 mm in both the x- and y-directions, using 50-μm increments. The experimentally measured alignment offsets were subsequently superimposed on the resulting numerical impedance map.

#### Temperature-dependent impedance simulations

For temperature-drift simulations, the electrical conductivity of the culture medium was defined as a temperature-dependent parameter following a linear relationship:

$$\sigma (\text{T})=\sigma ({\text{T}}_{0})[1+\alpha (\text{T}-{\text{T}}_{0})],$$

where σ(T_0_) is the conductivity measured at the reference temperature T_0_ and α is the relative temperature coefficient of conductivity. The coefficient was determined from the experimentally measured conductivity values obtained between 25 and 40 °C and was equal to α = 0.0179 °C^−1^ (1.79% °C^−1^). This linear approximation is consistent with previously reported conductivity measurements of tissue-culture media over a comparable temperature range [25]. Simulated impedance time courses were obtained by updating conductivity values according to the experimentally measured temperature evolution under incubator conditions. Heat transfer in solids was modelled using the Heat Transfer module to estimate the transient temperature equilibration of the culture chamber.

#### Flow and fluorescein transport simulations

Flow verification simulations were carried out using the Laminar Flow interface of the CFD module. The steady-state velocity field within the microfluidic circuit and culture chamber was computed under imposed volumetric flow rates, assuming incompressible Newtonian fluid behavior governed by the Stokes equations [26]. To account for pressure losses in the external perfusion circuit, simplified inlet and outlet capillary sections were initially included in the numerical geometry. Their effective hydraulic dimensions were selected using the Hagen–Poiseuille relationship to reproduce the hydraulic resistance of the perfusion circuit [26]. For the fluorescein transport simulations, this simplified representation was replaced by a more realistic geometrical representation of the upstream fluidic circuit, including the injector, capillary tubing, and inline Luer injector. Pressure losses associated with these components were therefore accounted for directly through the simulated fluidic geometry. No single lumped hydraulic-resistance value was imposed in this final geometrical model.

Fluorescein bolus transport was subsequently simulated using an advection-diffusion formulation [26]:

$$\partial \text{c}/\partial \text{t}+\text{u}\cdot \nabla \text{c}=\text{D}\nabla^{2}\text{c},$$

where c is the fluorescein concentration, u the velocity field obtained from the flow model, and D the diffusion coefficient of fluorescein in aqueous medium. A 100 μL bolus with initial concentration c_0_ = 0.47 mol·m^−3^ was introduced in the injection volume. Virtual regions of interest matching the experimental configuration (d = 4 mm separation) were defined within the culture chamber, and concentration time traces were extracted to determine the temporal shift Δt between regions. Mean flow velocities were derived from Δt and compared with experimental measurements (Supplementary Figure S2).

All numerical models used in this study are available in an online repository (see Data Availability Statement).

### Cell culture and 3D hydrogel preparation

#### Cell culture conditions

All cell cultures were maintained at 37 °C and 5% CO_2_ in a water-humidified incubator, corresponding to standard humidified cell-culture conditions. The human pancreatic ductal adenocarcinoma cell line HPAC (ATCC, Manassas, USA, reference CRL-2119™) was cultured in Dulbecco’s modified Eagle medium (1X DMEM; Gibco, Paisley, Scotland, reference 41965-039) supplemented with 10% (v/v) fetal bovine serum (FBS; Gibco, Paisley, Scotland, reference 10270-106), 2 mM L-glutamine (Gibco, Paisley, Scotland, reference 25030-024), and penicillin (100 IU·mL^−1^)-streptomycin (100 μg·mL^−1^) (Gibco, Paisley, Scotland, reference 15140-122). The HPAC cell line was genetically modified to express enhanced green fluorescent protein (HPAC-eGFP) (Vect’UB vectorology platform, TBM Core, UMS3427, University of Bordeaux).

#### Collagen I hydrogel preparation and 3D culture conditions

Rat tail collagen type I (BD Biosciences, reference 354236) was used for all 3D experiments. The collagen I stock solution had a concentration of 3.72 mg·mL^−1^. Collagen I stock solution and 1X DMEM were kept on ice prior to use to prevent premature gelation. Collagen I/1X DMEM mixtures were prepared in 1.5 mL tubes by mixing collagen I stock solution with 1X DMEM at defined volume ratios. No additional pH neutralization step was performed beyond dilution in 1X DMEM.

For collagen I concentration-dependent impedance measurements, mixtures containing 25%, 50%, 75%, and 100% (v/v) collagen I stock solution were prepared, corresponding to final collagen I concentrations of 0.93, 1.86, 2.79, and 3.72 mg·mL^−1^, respectively. For standard 3D culture experiments, collagen I and 1X DMEM were mixed at a 50:50 (v/v) ratio, corresponding to a final collagen I concentration of 1.86 mg·mL^−1^.

For extracellular matrix characterization and 3D cell culture, 50 μL of the collagen I/1X DMEM mixture was dispensed into each culture chamber using a micropipette while the device was placed on a cold plate. The device was then closed and transferred to an incubator (37 °C, 5% CO_2_) for 2 h to allow complete gel polymerization before impedance measurements.

For cell-laden hydrogels, HPAC-eGFP cells were trypsinized, counted using a Malassez counting chamber, and embedded in the collagen I/1X DMEM mixture at a final density of 1 × 10^6^ cells per 50 μL. The cell-laden hydrogel was loaded into the culture chambers following the same cold-handling and incubation procedure.

For long-term perfused 3D culture experiments, cell-laden collagen I hydrogels were perfused at 3.53 μL·min^−1^. Cells were cultured under continuous perfusion for 7 days. On day 7, 1 mL of a 5% (v/v) Triton X-100 solution (T-9284, Sigma-Aldrich, Missouri, USA) was added to the appropriate culture-medium reservoir to induce membrane permeabilization and cell death. Perfusion was then continued for an additional 2 days.

### Data processing and statistical analysis

#### Replication and data handling

All experiments were performed using independent microfluidic devices. Unless otherwise stated, (N) refers to the number of independent devices, whereas (n) refers to the total number of measured culture chambers or channels. For example, (N = 2), (n = 6) corresponds to measurements performed on three chambers or channels from each of two independent devices. Replication numbers are specified in the corresponding figure legends. Raw impedance data were exported as text files and processed into comma-separated values (CSV) format for analysis. Data are reported as mean ± standard deviation (SD), calculated from the measured chambers or channels, while the number of independent devices is indicated to document device-to-device replication. Chambers or channels measured within the same device were considered technical replicates.

#### Statistical analysis

Statistical analyses were carried out using GraphPad Prism 8.0.2 software. Comparisons involving more than two experimental conditions, including the effects of collagen I/1X DMEM volume and collagen I concentration, were performed using one-way ANOVA followed by Tukey’s multiple-comparison test. Comparisons between two independent groups, such as acellular collagen I hydrogels and HPAC-eGFP cell-laden collagen I hydrogels at a given time point, were performed using an unpaired two-tailed t-test. Comparisons between repeated measurements obtained from the same culture chambers at 2 and 24 h were performed using a paired two-tailed t-test.

Data are presented as mean ± standard deviation (SD). Differences were considered statistically significant for (p < 0.05). Independent experimental replicates correspond to independent microfluidic devices or chips, whereas technical replicates correspond to individual culture chambers or channels within a device.

### Ethical approval

No experiments involving human participants or live animals were performed in this study. The work used established commercial cell lines and commercially available collagen I. Therefore, no specific ethical approval was required.

## Results

The experimental validation was organized in three successive steps. First, we established the electrical and microfluidic operating conditions of the platform and characterized the effective impedance-sensing volume. Second, we quantified the influence of geometrical, thermal, and extracellular-matrix-related parameters on the measured impedance. Finally, we evaluated the ability of the platform to discriminate acellular from HPAC-eGFP cell-laden collagen I hydrogels under static and perfused conditions.

### Validation of the multichannel bioimpedance platform

#### Electrical measurement configuration and benefit of four-electrode impedance sensing

Impedance measurements were first compared using coplanar two-electrode and four-electrode configurations to evaluate electrode polarization effects. Figure 1a shows the electrode pairs selected within one culture chamber for two- and four-electrode measurements, while the complete 64-electrode biosensor layout and the allocation of eight electrodes per culture chamber are shown in Supplementary Figure S3. In phosphate-buffered saline (1X PBS), two-electrode measurements exhibited a strong low-frequency dispersion, with impedance magnitude increasing by more than one order of magnitude below 1 kHz and phase angle decreasing below −50° (Fig. 1b). In contrast, four-electrode measurements significantly reduced this dispersion, yielding quasi-frequency-independent impedance plateaus above ~2 kHz, whereas phase values remained within ±10° above approximately 2 kHz (Fig. 1b).

**Figure 1:**
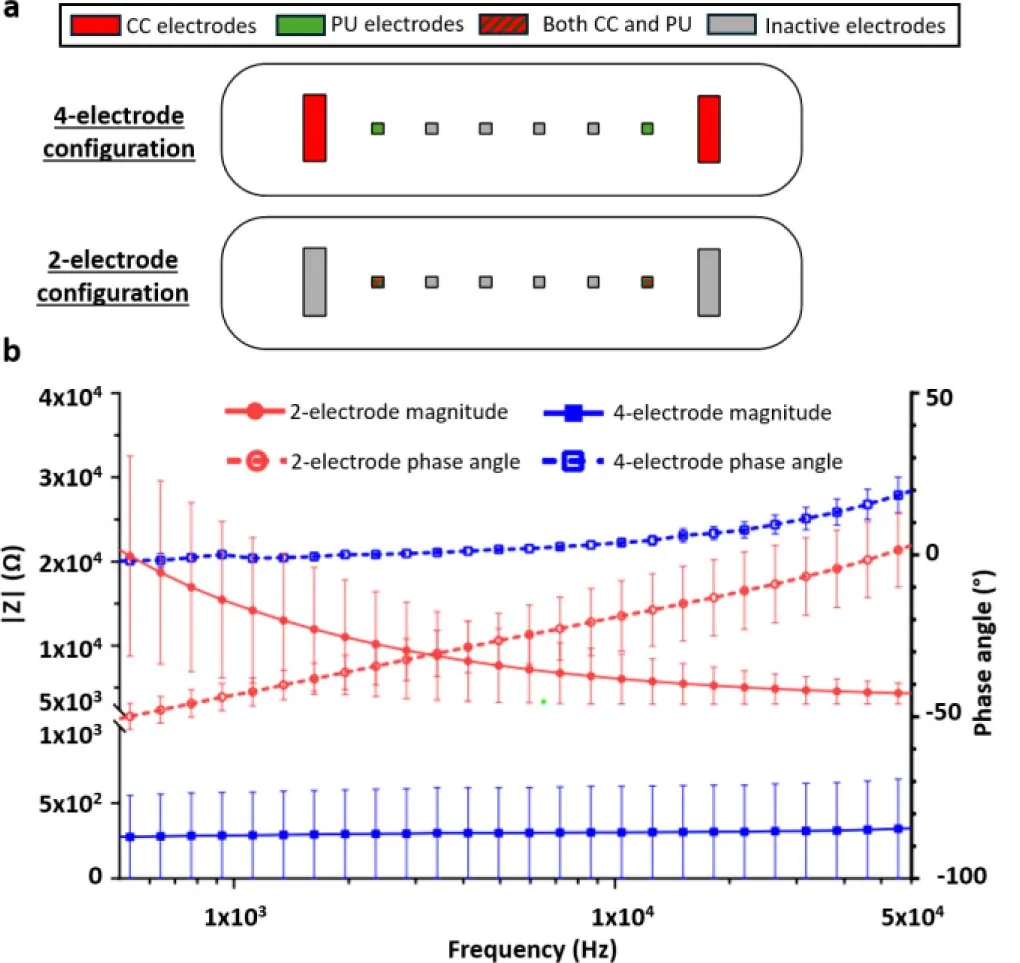
Comparison between two-electrode impedance and four-electrode transfer-impedance measurement configurations. (a) Schematic representation of the coplanar electrode layouts implemented within one tumor-on-chip culture chamber. In the four-electrode configuration, the outer current-carrying (CC) electrodes are used for current injection and the inner potential-sensing (PU) electrodes are used for voltage measurement. In the two-electrode configuration, the same electrode pair is used for both current injection and voltage sensing. Red electrodes indicate CC electrodes, green electrodes indicate PU electrodes, red/green hatched electrodes indicate electrodes used as both CC and PU electrodes, and grey electrodes indicate inactive electrodes. (b) Impedance magnitude and phase spectra measured in 1X phosphate-buffered saline (PBS) using the two-electrode impedance and four-electrode transfer-impedance configurations. The two-electrode configuration exhibits pronounced low-frequency dispersion due to electrode/electrolyte polarization, whereas the four-electrode configuration strongly reduces this contribution and provides a quasi-frequency-independent impedance plateau above approximately 2 kHz. In the four-electrode configuration, phase values remain within ±10° above approximately 2 kHz. The small positive phase observed at higher frequencies is consistent with tetrapolar transfer-impedance measurements and should not be interpreted as an inductive response of the electrolyte. Data are shown as mean ± SD from three channels measured on each of two independent devices (N = 2, n = 6).

In the four-electrode configuration, the measured quantity corresponds to a transfer impedance because current injection and voltage sensing are performed using spatially distinct electrode pairs. The small positive phase observed at higher frequencies should therefore not be interpreted as evidence of an inductive response of the electrolyte. Positive phase values can occur in tetrapolar transfer-impedance measurements of passive resistive-capacitive volume conductors as a consequence of the spatial distribution of current pathways and sensitivity fields [27–28].

These results demonstrate that the four-electrode configuration effectively reduces electrode polarization artefacts and provides a stable spectral window suitable for quantitative bioimpedance analysis in conductive liquids and hydrogel-based 3D cultures.

#### Verification of flow rates in the microfluidic circuit

Flow rates imposed by the peristaltic pump were verified experimentally using fluorescein bolus assays. A diluted fluorescein solution was injected into the microfluidic circuit, and its propagation through the culture chamber was monitored by fluorescence microscopy. The chamber geometry, flow direction, and positions of the two regions of interest (ROIs) used for analysis are shown in Supplementary Figure S2a,b.

Fluorescence intensity was extracted over time from each ROI, generating two time-dependent intensity profiles corresponding to the upstream and downstream positions within the chamber (Supplementary Figure S2c). The temporal delay between the two fluorescence signals was used to estimate the mean flow velocity in the culture chamber, as illustrated in Supplementary Figure S2d.

Measured flow velocities were then compared with numerical predictions over the investigated pump flow-rate range of 3–50 μL·min^−1^. Numerical simulations of fluorescein transport were performed using a geometrical representation of the upstream perfusion circuit that included the injector, capillary tubing, and in-line Luer injector, thereby accounting for the associated pressure losses. The simulated concentration profiles reproduced the experimentally observed temporal shift between the two ROIs, and the corresponding velocities were in good agreement with experimental estimates, with deviations below approximately 25% depending on the imposed flow rate and delay extraction method (Supplementary Figure S2e).

**Figure 2:**
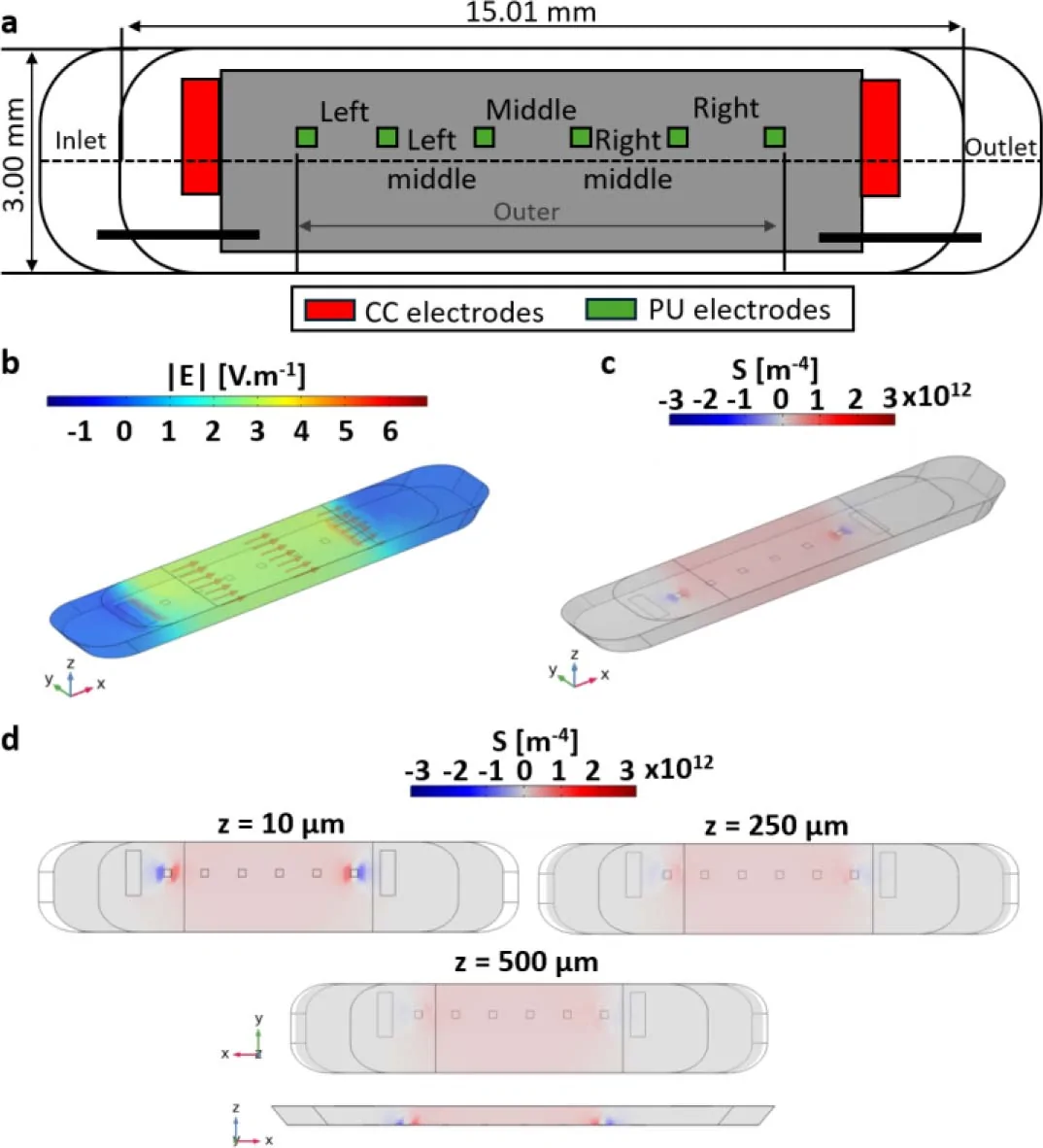
Sensitivity-field analysis of the four-electrode transfer-impedance measurement in a three-dimensional culture chamber. (a) Electrode geometry used in the numerical model. Red electrodes indicate current-carrying (CC) electrodes and green electrodes indicate potential-sensing (PU) electrodes. The layout comprises eight electrodes: two rectangular CC electrodes (1.50 × 0.50 mm) and six square PU electrodes (0.25 × 0.25 mm). All dimensions are given in mm. (b) Simulated electric-field magnitude distribution within the culture chamber. (c) Normalized sensitivity-field distribution highlighting the dominant contribution of the central chamber volume. (d) Sensitivity-field maps [m^−4^] in the xy plane at three vertical positions (z = 10, 250, and 500 μm), together with a representation along the z direction, illustrating the progressive decrease in sensitivity with increasing distance from the coplanar electrode plane and the reduced sensitivity to structures located in the upper part of the 3D culture volume.

Together, these results confirm that the imposed perfusion conditions were technically repeatable and consistent with numerical predictions based on laminar transport over the investigated flow-rate range.

#### Sensitivity-field analysis for 3D impedance measurements

Sensitivity-field simulations were performed to identify the effective measurement volume within the 3D culture chamber. Figure 2a illustrates the 3D geometry implemented in COMSOL Multiphysics, including electrode layout and chamber dimensions.

The simulated electric field magnitude revealed that current lines were primarily confined between the current-injecting electrodes, with negligible leakage outside the chamber volume (Fig. 2b). The corresponding normalized sensitivity field showed that more than 70% of the cumulative sensitivity was concentrated within the central region of the chamber (Fig. 2c). Axial cross-sections indicated a progressive decrease in sensitivity with increasing distance from the electrode plane, although this vertical decay was more clearly visualized when alternative, closer potential sensing electrode pairs were considered (Supplementary Figure S4). These complementary simulations showed that the sensitivity field remains strongly weighted toward the lower part of the chamber, near the coplanar electrode plane, and decreases toward the upper region of the hydrogel. This confirms that the selected four-electrode configuration is compatible with millimeter-scale collagen I hydrogels, but that impedance measurements pre-dominantly probe the region proximal to the electrodes, with reduced sensitivity to structures located in the upper part of the 3D culture volume.

#### Electrical validation and calibration of the impedance measurement system

Prior to investigating biological samples, the impedance measurement system was electrically validated using reference liquids with known conductivities. Three electrical conductivity standards (20, 30, and 50 mS·cm^−1^) and culture medium (1X DMEM) were characterized at room temperature (22.3 °C). Impedance spectra were acquired over the 500 Hz – 60 kHz frequency range using the four-electrode configuration, assuming homogeneous filling of the culture chamber (Fig. 3a).

All liquids exhibited stable and non-overlapping impedance magnitudes across the investigated frequency range, with impedance inversely proportional to electrical conductivity (Fig. 3a). Mean impedance values were 1480 ± 108 Ω, 943 ± 48 Ω, and 594 ± 47 Ω for the 20, 30, and 50 mS·cm^−1^ standards, respectively, whereas culture medium exhibited a higher impedance of 1780 ± 115 Ω.

**Figure 3:**
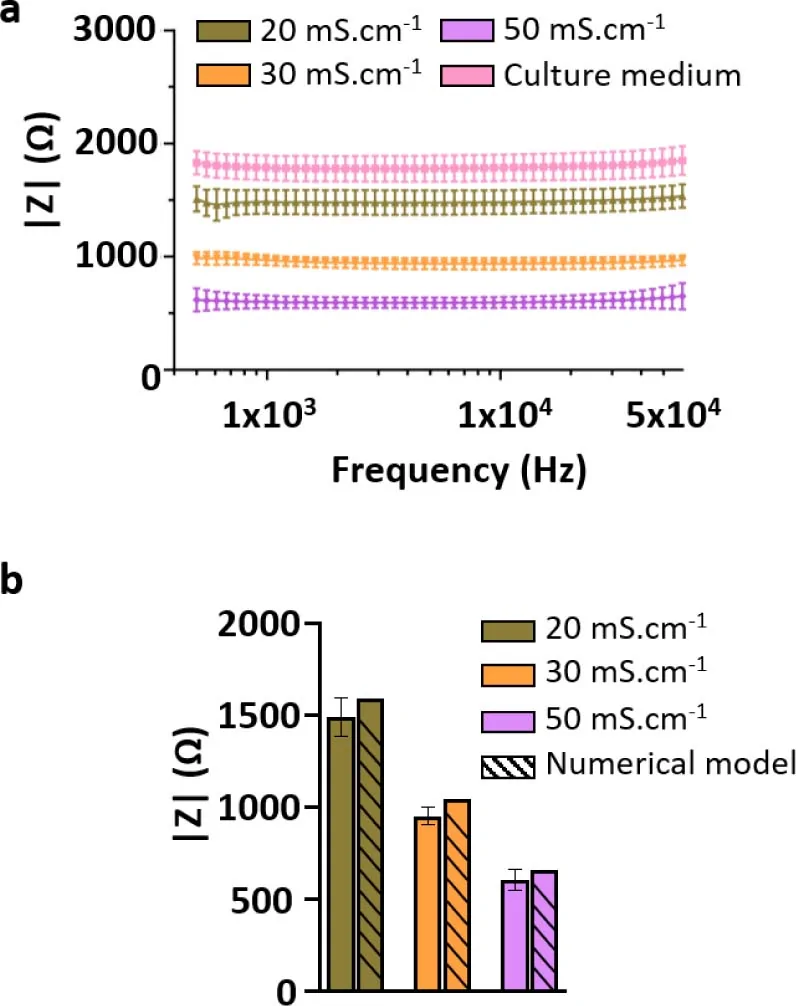
Electrical calibration and validation of the bioimpedance measurement system using reference liquids. (a) Impedance spectra measured for electrical conductivity standard solutions (20, 30, and 50 mS·cm^−1^) and culture medium (1X DMEM) at room temperature (22.3 °C), using the selected four-electrode configuration. The spectra exhibit distinct and non-overlapping impedance magnitudes across the investigated frequency range (500 Hz – 60 kHz), with impedance inversely proportional to solution conductivity. (b) Comparison between experimentally measured impedance values and numerical predictions obtained from three-dimensional COMSOL simulations for the same conductivity standards. Experimental values correspond to the frequency-averaged impedance magnitude, |Z_mean_|, computed over 500 Hz – 60 kHz, while numerical values were extracted from the simulated voltage difference between sensing electrodes. The relative deviation between experimental and numerical values remains below 10%, supporting the quantitative reliability of the impedance measurement system. Data are presented as mean ± SD from three independent devices and eight measured channels per device (N = 3, n = 24).

From the conductivity standards, an experimental cell constant of 2920 ± 79 m^−1^ was extracted, enabling quantitative conversion between impedance magnitude and effective electrical conductivity. Using this cell constant, the electrical conductivity of the culture medium was estimated at 16.4 mS·cm^−1^. Independent conductivity measurements showed values of 18.13 and 19.85 mS·cm^−1^ at 35 and 40 °C, respectively, corresponding by linear interpolation to approximately 18.8 mS·cm^−1^ at the physiological culture temperature of 37 °C. Thus, the expected conductivity at 37 °C lies within approximately 18.1 – 19.9 mS·cm^−1^, consistent with the reported temperature dependence of tissue-culture media [25].

To further validate the experimental measurements, impedance values for the conductivity standards were computed using a three-dimensional numerical model of the electrode and culture chamber geometry.

**Figure 4:**
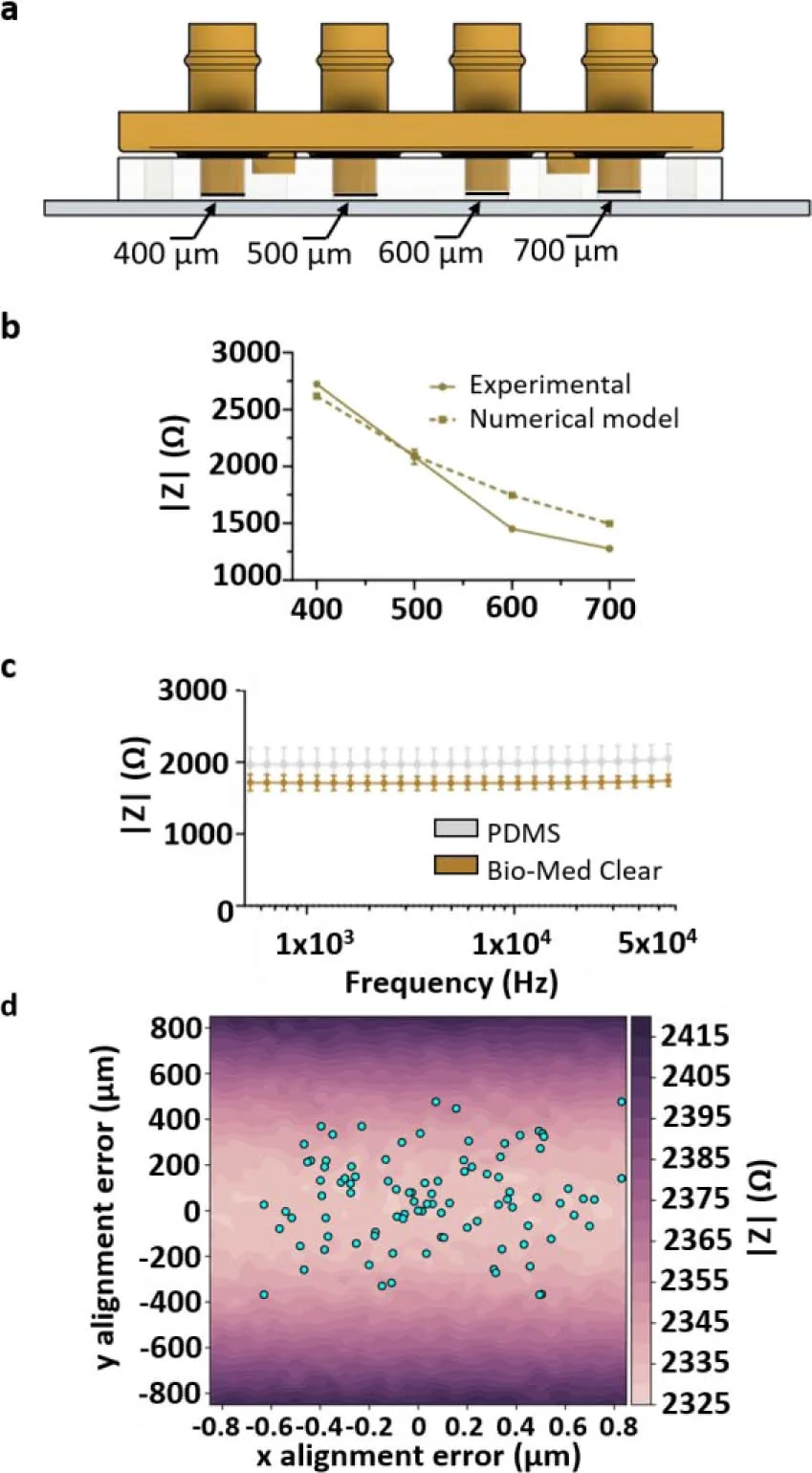
Influence of geometrical parameters on impedance measurements. (a) Schematic representation of the variable-height chamber configuration. Rigid closing caps were used to define four culture chamber heights within the same microfluidic system: 400, 500, 600, and 700 μm. (b) Experimental and numerical impedance magnitudes measured at 10 kHz as a function of culture chamber height. Increasing chamber height resulted in a systematic decrease in |Z|, in agreement with COMSOL predictions. Data are presented as mean ± SD from two independent devices and four measured chambers/channels (N = 2, n = 4). (c) Impedance magnitude spectra measured in 1X PBS using either a PDMS cap or a rigid 3D-printed Bio-Med Clear cap at room temperature. The two cap materials produced similar mean impedance spectra, indicating that cap material had only a minor influence on the average measured signal compared with chamber height. However, the rigid 3D-printed cap reduced measurement variability, consistent with improved mechanical control of chamber height compared with the softer PDMS cap. (d) Influence of lateral misalignment between the PDMS microfluidic layer and the electrode substrate on impedance magnitude. The color map represents simulated impedance magnitude as a function of x- and y-offsets. Dots indicate experimentally measured alignment offsets. Alignment offsets were measured from eight independent microfluidic devices and 64 culture chambers/channels (N = 8, n = 64).

Numerical simulations reproduced the experimentally observed trends, with simulated impedance values differing from experimental measurements by less than 10% (Fig. 3b). This discrepancy remained below 10% and was therefore smaller than the experimentally observed impedance changes associated with matrix and cellular contributions. Changes in collagen I composition produced impedance variations on the order of 30 – 40%, while increasing the loaded hydrogel volume from 60 to 100 μL resulted in an approximately 60% increase. Similarly, embedding HPAC-eGFP cells in collagen I decreased impedance magnitude at 10 kHz by approximately 48% at 2 h and 40% at 24 h compared with acellular controls.

The absence of significant frequency dependency for conductive reference liquids within the investigated range justified the use of impedance magnitude at a fixed frequency for time-resolved analyses. Based on the stable four-electrode impedance plateau observed experimentally, subsequent temporal and comparative measurements were therefore performed at 10 kHz.

### Physical and matrix-related determinants of impedance

#### Influence of geometrical parameters on impedance measurements

Chamber height was controlled using rigid closing caps defining the culture volume, while lateral alignment between the PDMS microfluidic layer and the electrode-bearing glass substrate was mechanically constrained and quantified during assembly.

The influence of geometrical parameters on impedance measurements was investigated experimentally and compared with numerical predictions. Increasing the chamber height from 400 μm to 700 μm resulted in a systematic decrease in impedance magnitude of approximately 35 – 45%, depending on frequency (Fig. 4a,b).

Experimental results were in good agreement with COMSOL simulations, with relative deviations below 10% across the tested geometries. A nearly linear dependence of impedance magnitude on chamber height was observed, with an average sensitivity of approximately 3 – 4 Ω·μm^−1^ at 10 kHz for the selected electrode configuration and conductivity standard.

Cap material had little influence on mean impedance, whereas the rigid 3D-printed cap reduced measurement variability compared with PDMS (Fig. 4c), supporting improved control of chamber geometry. Changing from a PDMS cap to the rigid 3D-printed cap decreased the frequency-averaged impedance magnitude from 1983 to 1713 Ω, corresponding to a relative decrease of approximately 13.6%. More importantly, measurement variability was substantially reduced, with the standard deviation decreasing from 281 to 94 Ω (approximately 66.5% reduction). This reduced variability is consistent with improved mechanical control of chamber height using the rigid cap. Lateral misalignment had only a limited influence on impedance magnitude (Fig. 4d). Numerical analysis yielded a sensitivity of approximately 0.0017 Ω·μm^−1^ to x-direction misalignment and 0.18 Ω·μm^−1^ to y-direction misalignment. The maximum impedance difference associated with the experimentally observed y-direction offsets was approximately 80 Ω, corresponding to less than approximately 1.5% of the measured impedance. Thus, chamber height remained the dominant geometrical contributor to impedance variability.

#### Temperature-induced impedance drift and stabilization time

The influence of temperature on impedance measurements was characterized under standard incubator conditions. The electrical conductivity of the culture medium increased approximately linearly between 25 and 40 °C, with an experimentally determined relative temperature coefficient of α = 0.0179 °C^−1^ (1.79% °C^−1^) (Fig. 5a).

Following placement of the device in a 37 °C incubator, impedance magnitude measured at 10 kHz decreased progressively over time (Fig. 5b). The total variation reached approximately 20 – 25% relative to the initial room-temperature value, and the signal reached a quasi-steady state after approximately 50 – 70 min, defining a characteristic thermal equilibration time.

The previously described COMSOL finite-element model, incorporating temperature-dependent conductivity, reproduced both the amplitude and the time scale of the experimentally observed impedance drift (Fig. 5b), confirming temperature as a first-order external factor influencing bioimpedance measurements in ToC systems.

#### Electrical characterization of collagen I hydrogels

The electrical contribution of the extracellular matrix was investigated using acellular collagen I hydrogels. First, the influence of collagen I concentration was evaluated at constant loaded volume. Impedance magnitude measured at 10 kHz increased monotonically with increasing collagen I concentration, indicating that the collagen matrix itself contributes measurably to the impedance signal (Fig. 6a).

Because the hydrogel is loaded before device closure, the amount of gel introduced into the chamber may influence the final matrix organization and compression state once the chamber is sealed. Increasing the loaded hydrogel volume from 60 μL to 100 μL, while keeping the final chamber geometry fixed, resulted in a marked increase in impedance magnitude (Fig. 6b).

This apparent loading-volume effect is therefore more likely associated with hydrogel compression and/or matrix reorganization during chamber closure than with a simple dependence on sample volume.

Impedance magnitude was higher for collagen I hydrogels than for culture medium, confirming that the matrix reduces the effective conductivity of the chamber volume (Fig. 6c).

Finally, impedance measurements performed using a 20 mS·cm^−1^ conductivity standard before collagen loading and after electrode cleaning yielded frequency-averaged impedance magnitudes of 1539 ± 61 Ω and 1560 ± 174 Ω, respectively, corresponding to a relative difference of approximately 1.4%, supporting the robustness of the cleaning protocol and the reusability of the electrode system (Fig. 6d). Additional time-resolved and frequency-resolved impedance measurements supporting the electrical characterization of collagen I hydrogels are provided in Supplementary Figure S5.

**Figure 5:**
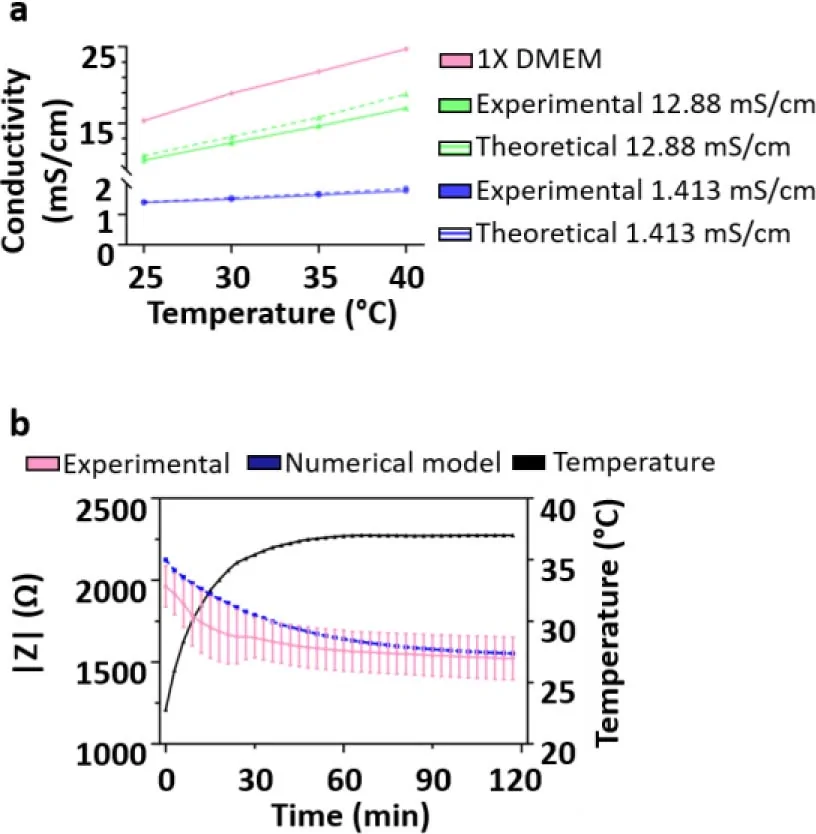
Temperature-induced drift of impedance measurements and stabilization time. (a) Electrical conductivity of the culture medium as a function of temperature, showing an approximately linear increase over the investigated range. Dotted lines represent the theoretical change in electrical conductivity values as given by the manufacturer. (b) Time-resolved impedance magnitude measured at 10 kHz following placement of the device in a 37 °C incubator. Experimental data are compared with numerical modelling incorporating temperature-dependent conductivity, illustrating the transient thermal equilibration and subsequent stabilization of the impedance signal. Data are presented as mean ± SD from two independent devices and four measured channels per device (N = 2, n = 8).

Together, these results demonstrate that the extracellular matrix is not a passive scaffold in impedance measurements, but a substantial contributor to the electrical signal through both its composition and its physical state within the chamber.

### Detection of cells embedded in 3D collagen I hydrogels

The capability of bioimpedance measurements to detect cells embedded within a 3D extracellular matrix was evaluated using HPAC-eGFP cell-laden collagen I hydrogels under static conditions. Collagen I hydrogels (50% v/v collagen I/1X DMEM, 50 μL) containing HPAC-eGFP cells were compared with acellular collagen I controls prepared under identical conditions.

Bioimpedance measurements were performed 2 and 24 h after gel polymerization. The frequency-averaged impedance magnitude of cell-free collagen I hydrogels showed no statistically significant difference between 2 and 24 h, with values of 3411 ± 891 Ω and 3103 ± 993 Ω, respectively (paired two-tailed t-test, p > 0.05; Fig. 7a). Similarly, no statistically significant difference was observed for HPAC-eGFP cell-laden hydrogels between 2 and 24 h, with values of 1806 ± 418 Ω and 1851 ± 259 Ω, respectively (paired two-tailed t-test, p > 0.05; Fig. 7b). However, a statistically significant difference was observed between acellular collagen I hydrogels and cell-laden hydrogels at both time points. At 2 h, impedance magnitude at 10 kHz decreased from 3430 ± 782 Ω in acellular collagen I to 1776 ± 455 Ω in cell-laden collagen I. A similar difference was observed after 24 h, with values of 3066 ± 937 Ω and 1825 ± 249 Ω, respectively (unpaired two-tailed t-test, p < 0.05; Fig. 7c).

Inter-chamber variability remained limited, with coefficients of variation below 7% across replicate chambers for both acellular and cell-laden hydrogels. These results demonstrate that, under static conditions, coplanar four-electrode impedance sensing enables non-destructive and reproducible electrical discrimination between acellular and HPAC-eGFP cell-laden 3D collagen I matrices. However, the absence of significant impedance evolution between 2 and 24 h indicates that short-term static culture did not produce a measurable time-dependent electrical change under the conditions tested.

Extended time-resolved measurements performed under continuous perfusion are presented in Supplementary Figure S6. Under dynamic conditions, cell-laden hydrogels initially exhibited lower impedance than acellular collagen I controls, consistent with the static measurements. During long-term perfusion, gradual cellular evolution observed by fluorescence microscopy was not associated with a reproducible impedance trajectory that could be unambiguously attributed to cell proliferation. In contrast, perfusion with Triton X-100 induced an increase in impedance toward the level measured in acellular collagen I controls. Although membrane disruption initially releases intracellular electrolytes, these soluble components are continuously transported out of the chamber by perfusion rather than accumulating within the sensing volume. The resulting impedance increase therefore reflects the loss of the cellular contribution and a progressive shift of the measured signal toward that of the acellular collagen I matrix.

## Discussion

This work provides a systematic electrical and geometrical analysis of bioimpedance measurements performed in microfluidic tumor-on-chip systems, with a specific focus on the transition from liquid-filled chambers to static and dynamic three-dimensional collagen-based cultures. Rather than treating impedance as a direct proxy for cell number or viability, our results show that the measured signal integrates multiple contributions, including electric field distribution, effective conductive volume, matrix composition, matrix physical state, and cellular content. Importantly, the cellular compartment remains detectable, as shown by the reproducible impedance decrease induced by HPAC-eGFP cells embedded in collagen I. However, under continuous perfusion, subtle time-dependent cellular changes are more difficult to interpret because they can be masked by matrix-related, geometrical, thermal, and conductive-volume contributions.

**Figure 6:**
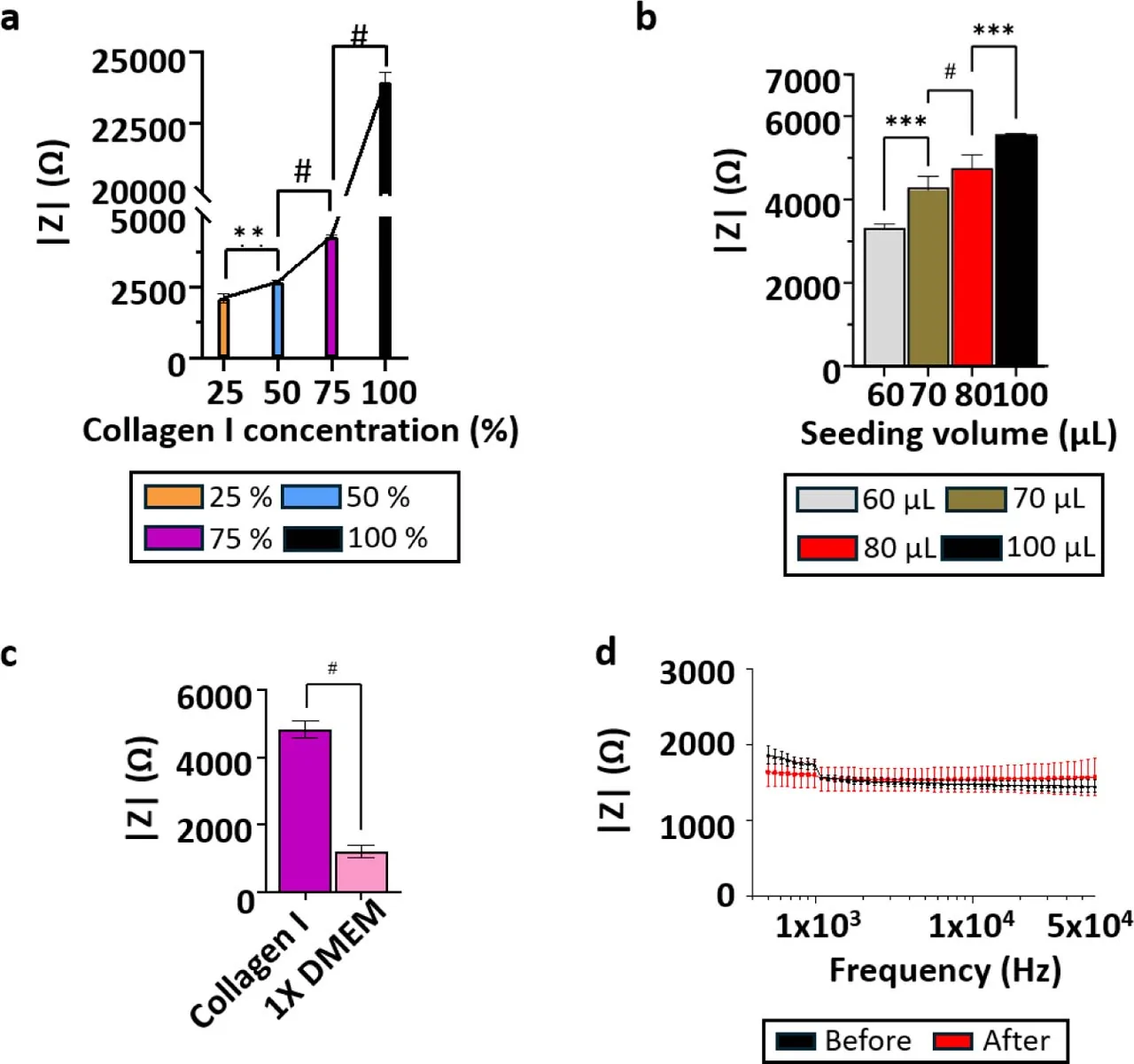
Electrical characterization of acellular collagen I hydrogels. (a) Relationship between collagen I concentration and impedance magnitude measured at 10 kHz at constant loaded volume. Collagen I/1X DMEM mixtures contained 25%, 50%, 75%, and 100% (v/v) collagen I stock solution, corresponding to final collagen I concentrations of 0.93, 1.86, 2.79, and 3.72 mg·mL^−1^, respectively. Data are presented as mean ± SD from three independent devices and six measured chambers/channels (N = 3, n = 6). (b) Relationship between loaded collagen I hydrogel volume and impedance magnitude measured at 10 kHz in a fixed chamber geometry, highlighting the influence of gel loading and compression on the measured signal. Data are presented as mean ± SD from three independent devices and six measured chambers/channels (N = 3, n = 6). (c) Comparison of impedance magnitude between culture medium and collagen I hydrogel, illustrating the higher impedance associated with the collagen matrix. Data are presented as mean ± SD from three independent devices and twelve measured chambers/channels (N = 3, n = 12). (d) Impedance measurements of a 20 mS·cm^−1^ conductivity standard before and after collagen loading and electrode cleaning, demonstrating measurement robustness and low signal variability. Data are presented as mean ± SD from three independent devices and twelve measured chambers/channels (N = 3, n = 12).

### Impedance as a volumetric, field-weighted measurement

Bioimpedance measurements performed in microfluidic systems inherently probe a weighted average of the electrical properties of the interrogated volume, as defined by the sensitivity field of the electrode configuration. This fundamental principle, formalised in electrical impedance tomography and reciprocity-based formulations, has been extensively discussed in the context of impedance imaging and microelectrode systems [29]. Our sensitivity-field simulations confirm that, for a coplanar four-electrode configuration, the electric field is strongly concentrated near the electrode plane and decays rapidly along the vertical axis. Therefore, impedance measurements in millimetre-scale 3D cultures remain dominated by the conductive volume proximal to the electrodes, particularly in coplanar electrode geometries.

These sensitivity-field effects may help explain differences between two-dimensional electrical cell-substrate impedance sensing (ECIS), which is highly sensitive to cell attachment and monolayer morphology [30], and three-dimensional impedance measurements, in which electrode geometry, scaffold properties, and the spatial distribution of cells can substantially influence the measured response [21,24]. This does not imply that cellular effects are absent in 3D systems, but rather that they are detected through a geometry-dependent sensitivity field. The comparable spectral behaviour observed across the six channels tested on two independent devices supports the reproducibility of the four-electrode configuration; however, the present dataset does not constitute an exhaustive validation of all 64 electrode channels, and channel-to-channel variability across the complete array was not systematically assessed.

**Figure 7:**
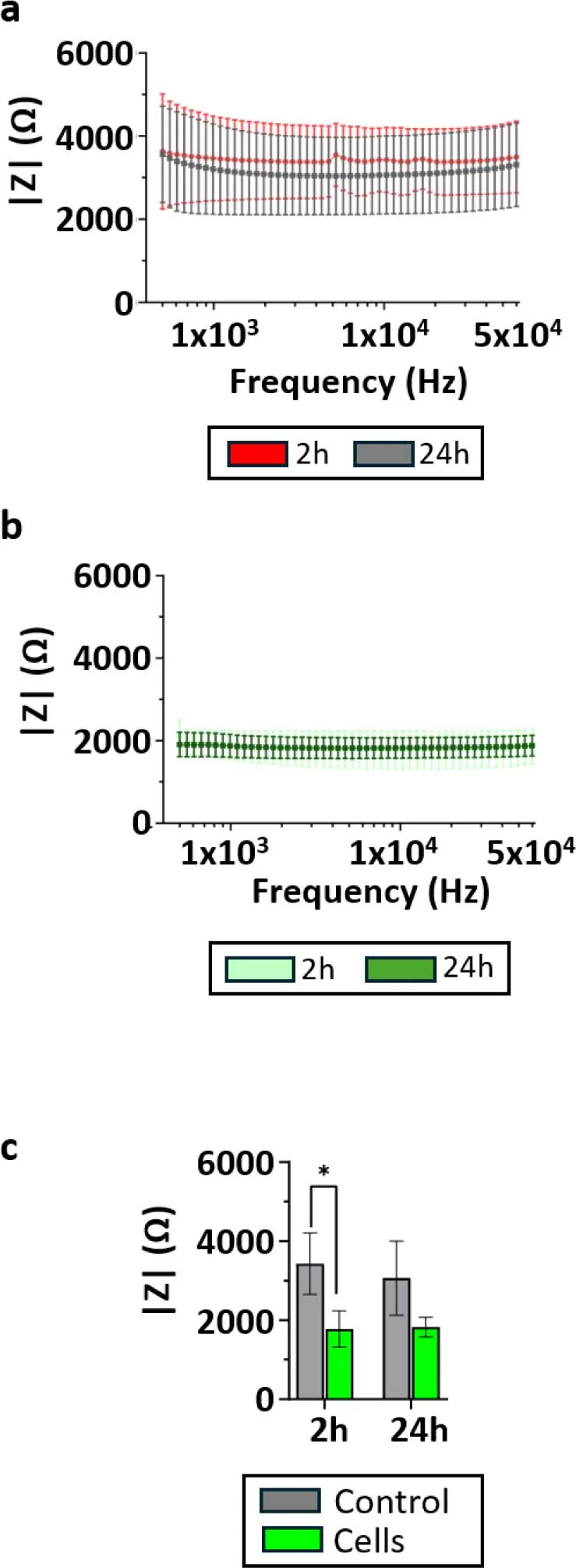
Detection of cells embedded in 3D collagen I hydrogels by bioimpedance measurements. (a) Impedance magnitude spectra measured for acellular collagen I hydrogels at 2 h and 24 h after gel polymerization. (b) Impedance magnitude spectra measured for HPAC-eGFP cell-laden collagen I hydrogels at 2 h and 24 h after gel polymerization. (c) Impedance magnitude extracted at 10 kHz for acellular and cell-laden collagen I hydrogels, showing a clear separation between the two conditions at both time points. Data are presented as mean ± SD from three independent devices and six measured chambers/channels (N = 3, n = 6). Comparisons between 2 and 24 h within each culture condition were performed using paired two-tailed t-tests. Comparisons between acellular and cell-laden hydrogels at each time point were performed using unpaired two-tailed t-tests. p < 0.05.

### Dominant role of geometry and chamber dimensions

Our experimental and numerical results clearly identify chamber height as the dominant geometrical parameter influencing impedance magnitude. Increasing the culture chamber height leads to a systematic decrease in impedance, with a sensitivity on the order of a few ohms per micrometre. This behaviour is consistent with recent microfluidic impedance studies that explicitly analysed channel dimensions as first-order design parameters [31,32].

Importantly, lateral misalignment between the micro-fluidic layer and the electrode substrate produced only minor impedance variations, confirming that volumetric effects dominate over planar positioning errors. This finding supports the robustness of the proposed ToC architecture for quantitative impedance studies, provided that chamber height is tightly controlled.

### Temperature and medium conductivity as first-order external factors

A key contribution of this work is the explicit characterisation of temperature-induced impedance drift under incubator conditions. We show that the electrical conductivity of culture medium increases approximately linearly with temperature, leading to a substantial decrease in measured impedance during thermal equilibration. This effect alone accounts for impedance variations comparable to, or larger than, those induced by changes in cell state or matrix composition.

While temperature effects on conductivity are well known in electrochemistry and geophysics [33] and are thoroughly described in electrochemical impedance spectroscopy literature [34], they are often overlooked or insufficiently controlled in bioimpedance studies. Our results demonstrate that, without explicit stabilization or correction, temperature-driven conductivity changes can mask biologically relevant signals, particularly in long-term or dynamic experiments. This issue is especially important in perfused tumor-on-chip experiments requiring repeated interventions, such as medium exchange, compound addition, or drug perfusion, which may transiently disturb the thermal equilibrium of the system. In practice, such perturbations can be minimized by pre-equilibrating media, drugs, reservoirs, and tubing at the experimental temperature, limiting incubator opening time, using in-line injection ports or automated switching systems, and allowing a defined re-equilibration period after each intervention before interpreting impedance changes. In addition, the use of acellular reference channels or simultaneous temperature monitoring may help distinguish true biological responses from temperature-induced conductivity drift.

### Cellular contribution and limits of dynamic impedance monitoring

The static cell-embedding experiments demonstrate that pancreatic cancer cells contribute measurably to the electrical response of collagen I-based 3D cultures. HPAC-eGFP cell-laden hydrogels exhibited a reproducible decrease in impedance magnitude compared with acellular collagen I controls at both 2 h and 24 h. This confirms that the integrated coplanar four-electrode sensor can discriminate cell-laden from acellular matrices under controlled static conditions.

However, the absence of significant impedance evolution between 2 h and 24 h indicates that the system did not detect short-term temporal changes in the cell/gel construct under static culture. This distinction is important: the sensor detects the presence of embedded cells as a baseline electrical difference, but this does not automatically translate into a quantitative readout of gradual cell proliferation or viability changes over time.

The analysis also supports the use of raw impedance magnitude rather than a normalized cell index. Cell-index formulations are useful in many ECIS studies, but there is no universal definition for 3D hydrogel-based systems. In such systems, the matrix itself can evolve through hydration, remodeling, or changes in effective conductivity. Normalization may therefore amplify or mask matrix-related variations and lead to misleading biological interpretations. Interpreting raw impedance magnitude allows the respective contributions of the matrix and cellular compartment to be considered more explicitly.

Under dynamic perfusion, gradual cell proliferation observed by fluorescence microscopy was not associated with a reproducible time-dependent impedance variation. The return of impedance toward the acellular collagen I baseline after Triton X-100 treatment further supports a measurable contribution of the cellular compartment. Under continuous perfusion, soluble intracellular electrolytes released upon membrane disruption are transported out of the sensing volume, preventing their accumulation from producing a sustained conductivity increase. Several factors may explain this limited dynamic sensitivity. First, the coplanar electrode geometry produces a sensitivity field that is strongly weighted toward the electrode plane and decreases along the vertical axis, reducing sensitivity to cellular changes occurring in the upper part of the hydrogel. Second, impedance changes in 3D cultures may depend not only on cell number, but also on cell spatial distribution, membrane integrity, cell-cell contacts, and the formation of electrically relevant intercellular pathways. Third, under perfusion, slow hydrogel hydration, remodeling, or partial mechanical degradation may modify the matrix contribution over time and mask subtle cell-dependent variations. Finally, connection to the microfluidic circuit increases the effective conductive volume, thereby reducing the relative weight of cellular changes in the global impedance measurement.
